# Recurrence of Postneurosurgical Central Nervous System Infection Caused by *Acinetobacter baumannii* in a Critically Ill Patient Treated With Cefiderocol. A Case Report

**DOI:** 10.1155/crdi/7327571

**Published:** 2026-04-19

**Authors:** Giuseppe Accurso, Angelica Ancona, Giovanni Marco Costa, Filippo Vitale, Domenico Petronaci, Giorgia Corpora, Luigi Profera, Antonino Giarratano, Santi Maurizio Raineri

**Affiliations:** ^1^ Department of Anaesthesia, Intensive Care and Emergency, Policlinico Paolo Giaccone, Palermo, Sicily, Italy; ^2^ Department of Neuroscience and Emergency With Trauma Center, A.O.O.R. Villa Sofia Cervello, Palermo, Sicily, Italy; ^3^ Department of Precision Medicine in Medical, Surgical and Critical Care, University of Palermo, Palermo, Sicily, Italy, unipa.it

**Keywords:** *Acinetobacter baumannii*, cefiderocol, central nervous system infections, meningitis, multidrug-resistant bacteria

## Abstract

**Introduction:**

*Acinetobacter baumannii* (*A. baumannii*), a gram‐negative bacterium, has become a significant hospital pathogen and the cause of severe nosocomial infections, especially in the intensive care unit (ICU). Bacterial meningitis is a recognized complication that can occur after neurosurgery, and the number of cases of multidrug‐resistant (MDR) meningitis caused by *A. baumannii* is increasing. Critically ill patients in the ICU are at high risk, and the consequences are potentially fatal. Cefiderocol is a novel cephalosporin that can treat carbapenem‐resistant infections in patients with limited treatment options. No clinical trials have investigated its capability to penetrate cerebrospinal fluid.

**Case Presentation:**

We describe the case of a 66‐year‐old man who was admitted to our neurointensive care unit after head trauma. On the CT scan performed on admission to the hospital, there was evidence of a subdural hematoma and subarachnoid and intraparenchymal hemorrhages in the frontotemporal region bilaterally. The patient then underwent a decompressive craniectomy. During hospitalization and following cranioplasty, the patient experienced a central nervous recurrence of carbapenem‐resistant *A. baumannii* (CRAB) meningitis, which was treated with cefiderocol, resulting in a resolution of signs and symptoms of infection within a few days.

**Conclusion:**

We present the first case of CRAB meningitis treated with this cefiderocol dosing regimen. In our case of postneurosurgical MDR meningitis reinfection caused by *A. baumannii*, cefiderocol as monotherapy brought about a clinical and microbiological cure in both blood and cerebrospinal fluid without the occurrence of adverse effects such as seizure.

**Highlights**
• *Acinetobacter baumannii* (*A. baumannii*), a gram‐negative bacterium, is a significant hospital pathogen and a cause of severe nosocomial infections.
• Bacterial meningitis is a recognized complication that can occur after neurosurgery, particularly in critically ill patients.
• Cefiderocol is a novel cephalosporin that treats carbapenem‐resistant infections in patients with limited treatment options.
• Postneurosurgical MDR meningitis reinfection caused by *A. baumannii* can be treated with cefiderocol monotherapy without adverse effects.

## 1. Introduction

*Acinetobacter* *baumannii*​ (*A. baumannii*), a gram‐negative bacterium, has emerged as a significant hospital pathogen, particularly in intensive care units (ICUs), where it causes severe nosocomial infections [1].

### 1.1. Postneurosurgical Meningitis and Clinical Burden

Bacterial meningitis is a recognized postneurosurgical complication, with an increasing proportion of cases caused by multidrug‐resistant (MDR) *A. baumannii* [2].

Critically ill ICU patients are at high risk for such infections, which often have potentially fatal outcomes. Most reported cases of *A. baumannii* meningitis have been associated with external ventricular drainage (EVD), cerebrospinal fluid (CSF) leaks, or head trauma. Central nervous system (CNS) infections pose unique challenges due to variable antibiotic penetrability and potentially suboptimal CSF antibiotic concentrations with standard dosing regimens [3].

While gram‐positive bacteria such as staphylococci and resistant gram‐negative bacilli are commonly implicated in postsurgical infections, the treatment of nosocomial CNS infections often relies on carbapenems and glycopeptides, sometimes in combination with intrathecal administration of colistin, polymyxin B, or aminoglycosides [2]. Intravenous meropenem combined with intraventricular aminoglycosides is considered a superior regimen for suspected *A. baumannii* meningitis. For carbapenem‐resistant *A. baumannii* (CRAB) infections, polymyxins are frequently utilized, often in combination with intraventricular antibiotics and the removal of infected neurosurgical devices. However, the emergence of MDR, extensively drug‐resistant (XDR), and pan‐drug‐resistant (PDR) bacteria poses significant challenges to manage severe CNS infections [4].

Cefiderocol, a novel siderophore cephalosporin, demonstrates a unique mechanism of action by exploiting bacterial iron transport systems to facilitate drug uptake, thereby achieving high stability against β‐lactamase hydrolysis, including extended‐spectrum β‐lactamases and carbapenemases (e.g., KPC, NDM, VIM, and OXA‐48‐like) [5, 6].

### 1.2. Cefiderocol and Therapeutic Rationale

This antibiotic has shown potent in vitro activity against various gram‐negative bacilli, including Acinetobacter spp., and is approved in Europe for treating infections caused by aerobic gram‐negative bacteria in adults with limited treatment options [7]. However, data on cefiderocol penetration into the CSF remain limited and derive mainly from animal models and a small number of human pharmacokinetic reports demonstrating measurable CSF concentrations during CNS infections. In a rat meningitis model, cefiderocol demonstrated increased CSF penetration in the presence of meningeal inflammation [8].

## 2. Case Description

**FIGURE 1:**
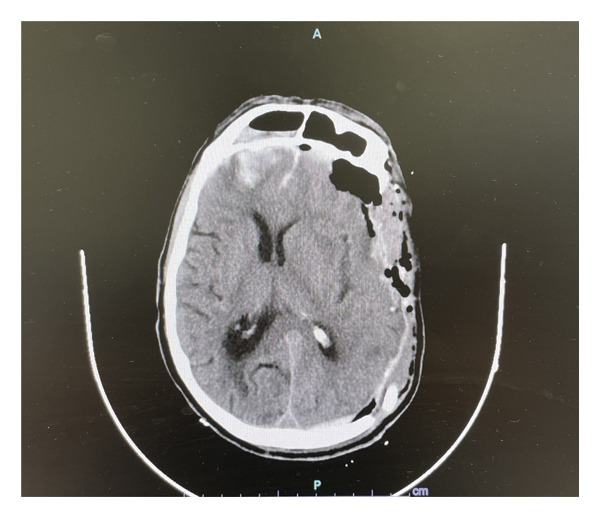
A CT scan after decompressive craniectomy.

We report the case of a 66‐year‐old male admitted to the neurointensive care unit following head trauma. A CT scan upon admission revealed a subdural hematoma in the left occipital‐parietal region with a 1.9 cm midline shift, along with subarachnoid and intraparenchymal hemorrhages bilaterally. The patient underwent decompressive craniectomy in December 2022 (Figure 1). During the initial postoperative period, there were no clinical or radiological signs of hydrocephalus, no EVD was required, and there was no suspicion of CNS infection.

In March 2023, acute hydrocephalus prompted the insertion of an external ventricular shunt.

Five days later, CSF cultures revealed non‐MDR *A. baumannii* (Table 1). The shunt was replaced, and a 14‐day treatment with levofloxacin (750 mg) and intrathecal colistin (125,000 IU every 24 h) was initiated.

**TABLE 1:** The antibiotic susceptibility test for *Acinetobacter baumannii* shown below has been interpreted in accordance with the EUCAST criteria.

Antibiotic name	MIC (μg/mL)	Interpretation
Amikacin	8	Susceptible
Ciprofloxacin	0.125	Inducible
Colistin	≤ 0.5	Susceptible
Gentamicin	2	Susceptible
Imipenem	> 8	Resistant
Levofloxacin	≤ 0.25	Susceptible
Meropenem	> 16	Resistant
Tobramycin	2	Susceptible
Trimethoprim/sulfamethoxazole	≤ 1/19	Susceptible

*Note:* March 2023.

In April, after two negative CSF cultures and resolution of infection symptoms, ventriculoperitoneal shunt placement and cranioplasty were performed.

In May, the patient presented with hemodynamic instability, elevated inflammatory markers, and fever > 39°C. Blood and CSF cultures confirmed CRAB using T2 magnetic resonance followed by traditional culture methods (Table 2). Antibiograms were interpreted per EUCAST criteria (Version 13.0).

**TABLE 2:** The antibiotic susceptibility test for *Acinetobacter baumannii* multidrug resistant (MDR) shown below has been interpreted in accordance with the EUCAST criteria.

Antibiotic name	MIC (μg/mL)	Interpretation
Ciprofloxacin	> 1	Resistant
Colistin	≤ 0.5	Susceptible
Gentamicin	> 4	Resistant
Imipenem	> 8	Resistant
Levofloxacin	> 1	Resistant
Meropenem	> 16	Resistant
Tobramycin	> 4	Resistant
Trimethoprim/sulfamethoxazole	> 4/76	Resistant
Cefiderocol	0.32	Susceptible

*Note:* April 2023.

A multidisciplinary team evaluated the risks and benefits of replacing the ventriculoperitoneal shunt and removing the cranioplasty. Given the patient’s severe neurological condition, bloodstream infection (BSI), and meningitis, intravenous cefiderocol (2 g over 3 h every 6 h) was initiated. This regimen accounted for hyperfiltration (creatinine median: 0.4 mg/dL; eGFR: 120 mL/min/1.73 m^2^).

**FIGURE 2:**
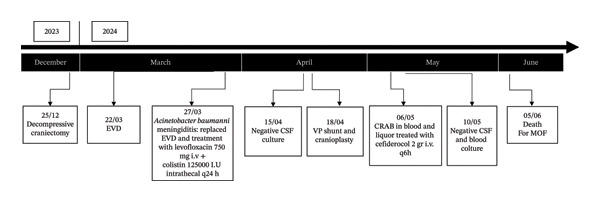
Timeline of the event. EVD: external ventricular drainage; VP: ventriculoperitoneal; CRAB: carbapenemase‐resistant *Acinetobacter baumannii*; MOF: multiorgan failure.

Clinical and laboratory improvements were evident within two days. Blood and CSF cultures taken on Days 5 and 10 were negative. The patient completed a 14‐day course of cefiderocol without adverse effects. Unfortunately, in June, the patient succumbed to multiorgan failure unrelated to infection (Figure 2).

## 3. Discussion

This is the first reported case of CRAB meningitis treated with cefiderocol monotherapy at this dosing regimen.

Postneurosurgical meningitis caused by *A. baumannii* is a severe infectious complication requiring timely diagnosis and appropriate therapy. Cefiderocol’s unique pharmacodynamics make it a promising option for treating CRAB infections, particularly in CNS sites where conventional antibiotics may fail due to limited penetration [9, 10].

Data on cefiderocol penetration into the CSF remain limited. Available evidence derives mainly from animal models and a small number of human pharmacokinetic reports. In a rat meningitis model, cefiderocol demonstrated increased CSF penetration in the presence of meningeal inflammation, suggesting that blood–brain barrier disruption may enhance drug delivery to the CNS [8]. In humans, measurable CSF concentrations have been reported during treatment of CRAB meningitis, with CSF‐to‐plasma ratios broadly consistent with other β‐lactams under inflammatory conditions [6]. These findings support the biological plausibility of cefiderocol efficacy in CNS infections, although optimal dosing strategies and target attainment in CSF remain to be fully elucidated.

Clinical trials such as CREDIBLE‐CR have demonstrated cefiderocol’s efficacy in treating carbapenem‐resistant infections, supporting its use in patients with limited options [11]. Additional real‐world evidence has described favorable outcomes with cefiderocol in difficult‐to‐treat gram‐negative infections, including nosocomial BSIs. These findings are consistent with the clinical response observed in our patient [12].

Despite its promise, challenges remain in establishing optimal dosing regimens and managing resistance emergence [13].

Our patient’s case highlights cefiderocol’s potential to achieve clinical and microbiological cure in CNS infections, even without adjunctive intrathecal therapy. Nonetheless, limitations such as the lack of CSF antibiotic concentration measurements in our report underscore the need for further research to optimize cefiderocol use.

## 4. Conclusion

Cefiderocol is a valuable addition to the therapeutic arsenal against MDR *A. baumannii* infections. In our case, cefiderocol monotherapy resulted in the clinical and microbiological cure of CRAB meningitis without adverse effects. Further studies must refine its clinical utility and ensure its effectiveness through prudent stewardship practices.

## Author Contributions

Giuseppe Accurso and Angelica Ancona contributed to conceptualization and writing of the original draft. Giovanni Marco Costa, Giorgia Corpora, and Santi Maurizio Raineri contributed to methodology, data curation, and drafting of the manuscript. Santi Maurizio Raineri, Domenico Petronaci, Luigi Profera, and Filippo Vitale contributed to investigation and visualization. Santi Maurizio Raineri and Antonino Giarratano supervised the project. Giuseppe Accurso, Santi Maurizio Raineri, Giorgia Corpora, and Antonino Giarratano reviewed and edited the final manuscript.

## Funding

This research did not receive any specific grant from funding agencies in the public, commercial, or not‐for‐profit sectors.

## Disclosure

This work has not been deposited as a preprint and has not been previously presented at any scientific conference or seminar. All authors read and approved the final version of the manuscript.

## Ethics Statement

Ethical approval was not required for this single‐patient case report, in accordance with institutional and national regulations, as the report does not contain identifiable personal information.

## Consent

Written informed consent for publication of this case report and accompanying images was obtained from the patient’s next of kin. A copy of the consent form is available for review by the Editor‐in‐Chief upon request.

## Conflicts of Interest

The authors declare no conflicts of interest.

## Supporting Information

The authors have completed the CARE Checklist for case reports, and the completed checklist is submitted as supporting information.

## Supporting information


**Supporting Information** Additional supporting information can be found online in the Supporting Information section.

## Data Availability

No datasets were generated or analyzed for this case report. All relevant clinical details are included within the manuscript.
